# Phytochemical profiling and seasonal variation of essential oils of three *Callistemon species* cultivated in Egypt

**DOI:** 10.1371/journal.pone.0219571

**Published:** 2019-07-11

**Authors:** Haidy A. Gad, Iriny M. Ayoub, Michael Wink

**Affiliations:** 1 Pharmacognosy Department, Faculty of Pharmacy, Ain Shams University, Cairo, Egypt; 2 Institute of Pharmacy and Molecular Biotechnology, Heidelberg University, INF, Heidelberg, Germany; Embrapa Agroindústria Tropical, BRAZIL

## Abstract

The genus *Callistemon* comprises evergreen shrubs or small trees, widely cultivated as ornamentals and for essential oil production. *Callistemon* is well-recognized in folk medicine for its anti-cough, anti-bronchitis, and insecticidal activities. In the current study, we profiled the essential oil composition of the leaves of *C*. *citrinus*, *C*. *rigidus* and *C*. *viminalis* (Myrtaceae) collected during different seasons by GLC-MS coupled to multivariate data analysis. Antioxidant, anti-inflammatory and anti-proliferative activities of *Callistemon* essential oils were evaluated. A total of 29 compounds were tentatively identified. Oxygenated monoterpenes dominated in essential oils, where eucalyptol represented the major constituent in the three *Callistemon* species in all seasons. Multivariate data analysis including Principal Component Analysis (PCA) and Hierarchical Cluster Analysis (HCA) were applied to discriminate between different *Callistemon* species in each season and to investigate any correlation between the metabolic profile of each species within different seasons. As expected, PCA plot could discriminate the three *Callistemon* species in the four seasons. The dendrogram from HCA confirmed the results of PCA as it showed the same segregation pattern regarding the discrimination of different *Callistemon* species. *C*. *viminalis* showed more pronounced antioxidant activity than *C*. *citrinus*, exhibiting IC_50_ values of 1.40 mg/mL and 1.77 mg/mL, respectively. Meanwhile, *C*. *rigidus* showed very weak antioxidant activity. All oils showed membrane stabilization activity in hypotonic solution induced haemolysis assay, where *C*. *viminalis* showed potent membrane stabilizing activity exhibiting IC_50_ value of 25.6 μg/mL comparable to that of the standard drug, indomethacin (17.02 μg/mL). Nevertheless, Callistemon essential oils were not cytotoxic in HCT-116 and Hela human cancer cell lines.

## Introduction

The genus *Callistemon* (Myrtaceae) consists of evergreen shrubs or small trees. It is widely used for essential oil production, and as ornamental, wind-breaking and degraded-land reclamation plants [1]. *Callistemon* originates from Australia, but is cultivated and introduced to most parts of the world [2]. *Callistemon* species possess attractive lanceolate leaves and characteristic 'bottlebrush' shaped flower spikes with prominent red stamens, thus, commonly named Bottlebrush [3]. The most widely cultivated member of this genus is *C*. *citrinus*, commonly known as Crimson or Lemon Bottlebrush [2]. *C*. *viminalis* is a small tree or shrub characterized by pendulous foliage and thus, was given the name Weeping Bottlebrush [2]. *C*. *rigidus*, commonly called Stiff or Erect Bottlebrush, is a stiff and upright shrub with red flower spikes [4].

*Callistemon* species produce essential oils. In folk medicine, *Callistemon* is well recognized for its insecticidal, as well as its anti-cough and anti-bronchitis activities [5]. The essential oils possess antimicrobial, antifungal, insecticidal, anthelmintic, antioxidant, anti-inflammatory and anti-nociceptive activities [5–8]. In China, *Callistemon* species, *mainly C*. *viminalis* is used in Traditional Chinese Medicine for treating hemorrhoids [9]. 1,8-Cineole figured as the predominant constituent reported in *Callistemon* essential oils. Other reported secondary metabolites include *α*-pinene, *β*-pinene, limonene, linalool, myrcene, and menthyl acetate [3]. The biological activity of an essential oil could not be ascribed to a single component but rather to the synergistic effects of the phytocomplex. This chemical complexity contributes to its biological activity, since each constituent of the phytocomplex is involved in the overall activity or may modulate the effects of the other constituents [10].

The main goal of this study is phytochemical profiling of essential oils from three *Callistemon* species (*C*. *citrinus*, *C*. *rigidus and C*. *viminalis*) in different seasons (spring, summer, autumn and winter) by GLC/MS. Principal component analysis (PCA) and hierarchical cluster analysis (HCA) were applied as pattern recognition techniques to discriminate between species and seasons. Moreover, essential oils were assessed for their antioxidant, anti-inflammatory and anti-proliferative activities.

## Materials and methods

### Plant material

Leaves of *Callistemon citrinus*, *C*. *rigidus and C*. *viminalis* (Myrtaceae) were collected in four different seasons from a private botanical garden in Giza, Egypt. Plants were kindly identified by Mrs. Trease Labib, Plant Taxonomy Consultant at the Ministry of Agriculture and former director of Orman Botanical Garden, Giza, Egypt. Voucher specimens were deposited in the herbarium of Pharmacognosy Department, Faculty of Pharmacy, Ain Shams University (PHG-P-CC; PHG-P-CR; and PHG-P-CV).

### Isolation of the essential oil

The essential oils from fresh leaves (100 g) of *Callistemon citrinus*, *C*. *rigidus and C*. *viminalis* were obtained by hydro-distillation for 4 h using a Clevenger-type apparatus. The oil was collected, dried over anhydrous sodium sulfate, weighed and kept in sealed vials at 4°C for further analyses. The yield in % (w/w) was determined in triplicate, based on the initial plant weight.

### GLC/MS analysis

Mass spectra were recorded using a Shimadzu GCMS-QP2010 (Kyoto, Japan) equipped with a split–splitless injector. Separation was achieved using an Rtx-5MS fused silica capillary column (30 m x 0.25 mm i.d. x 0.25 μm film thickness) (Restek, USA). The initial column temperature was kept at 45°C for 2 min and programmed to 300°C at a rate of 5°C/min, and kept constant at 300°C for 5 min. Injector temperature was 250°C. Helium was used as a carrier gas with a flow rate of 1.41 mL/min. Mass spectra were recorded applying the following conditions: Ion source temperature, 200°C; ionization voltage, 70 eV; (equipment current) filament emission current, 60 mA. Split mode injection of diluted samples (1% v/v) was applied with split ratio 1: 15.

### Identification of essential oil components

Essential oil components were tentatively identified by comparison of their mass spectra and retention indices with those listed in NIST Mass Spectral Library, 2011; Wiley Registry of Mass Spectral Data 8^th^ edition; and data reported in literature [11–13].

### Chemometric analysis

GLC/MS phytochemical profiling was subjected to chemometric analysis. Principal Component Analysis (PCA) comprises a first step in data analysis in order to provide an overview of all observations and samples to identify and evaluate groupings, trends and strong outliers [14, 15]. Hierarchal Cluster Analysis (HCA) was then applied to allow clustering of different *Callistemon* species. The clustering patterns was constructed by applying the complete linkage method used for group building; this representation is more efficient when the distance between clusters is computed by Euclidean method [14, 15]. For PCA and HCA, Unscrambler X 10.4 from CAMO (Computer Aided Modeling, AS, Norway) was employed.

### Antioxidant activity

Antioxidant activity of *Callistemon* essential oils was investigated using 1,1-diphenyl-2-picrylhydrazyl (DPPH) radical scavenging assay [16]. An aliquot (40 μL) of various concentrations of the essential oils (0.04–5.12 mg/mL) was added to 0.004% w/v DPPH in methanol (3 mL). Absorbance was recorded immediately against a blank using a UV-visible spectrophotometer (Milton Roy, Spectronic 1201). The decrease in absorbance at 515 nm was determined continuously, with the data being recorded at 1 min intervals until the absorbance was stabilized (16 min). Ascorbic acid was used as a reference compound. All experiments were performed in triplicates. The inhibition percentage (I%) was calculated according to the following equation:

$$Inhibition\%=\left[\left(A_{blank}‐A_{sample}\right)/A_{blank}\right]\;x\;100$$

Where A_blank_ = Absorbance of the blank (non-reduced DPPH) at t = 0 min and A_sample_ = absorbance of the test sample at t = 16 min.

### Anti-inflammatory activity

Membrane stabilization assay was used to assess the *in vitro* anti-inflammatory activity of *Callistemon* essential oils using hypotonic solution-induced erythrocyte hemolysis described by Shinde *et al*. [17].

#### Preparation of erythrocyte suspension

Whole blood was collected from rats via cardiac puncture under ether anesthesia into heparinized tubes. Blood was washed three times with 0.9% saline. The volume of saline was measured and reconstituted with isotonic buffer solution (pH 7.4) composed of 154 mM NaCl in 10 mM sodium phosphate buffer (pH 7.4) as 40% v/v suspension. The blood was then centrifuged at 3000 rpm for 10 minutes [17].

#### Hypotonic solution-induced hemolysis

Membrane stabilization activity of the essential oils was assessed using hypotonic solution-induced erythrocyte hemolysis [17]. Briefly, 0.5 mL of stock erythrocyte (RBCs) suspension was mixed with 5 mL of hypotonic NaCl solution (50 mM) in 10 mM sodium phosphate buffered saline (pH 7.4) containing the tested essential oil at a concentration of 7.81–1000 μg/mL. The control sample was composed of 0.5 mL of RBCs mixed with 5 mL hypotonic-buffered saline solution alone. Mixtures were incubated at room temperature for 10 min, then centrifuged at 3000 rpm for 10 min. Indomethacin was used as a reference standard. In 96 well plates, the absorbance (O.D.) of the supernatant was measured at 540 nm. The percentage inhibition of hemolysis or membrane stabilization percentage was calculated according to the method described by Shinde et al. [17]. The IC_50_ value was defined as the concentration of the sample that inhibited 50% erythrocyte hemolysis under the assay conditions.

$$\%Inhibition\;of\;Hemolysis\;(Or\%Membrane\;Stabilization\%)=100\;x\;\left(OD_{1}‐OD_{2}/OD_{1}\right)$$

Where,

OD_1_ is the optical density of the hypotonic-buffered saline solution alone

OD_2_ is the optical density of the test sample in hypotonic solution.

#### Anti-proliferative activity

The anti-proliferative activity of the essential oils was assessed against HCT-116 and Hela human cancer cell lines using MTT assay [13, 18]. Exponentially growing cells were seeded at a density of 10×10^4^ cells/well (HCT-116) and 15 x10^4^ cells/well (Hela) in 96-well plates. Stock solutions of essential oils in dimethyl sulfoxide (DMSO) were prepared. Essential oils were subjected to two-fold serial dilutions in the respective media where the maximal concentration of DMSO did not exceed 1%. Doxorubicin was used as a positive control. Cells were treated with 100 μL of the tested essential oils at concentrations ranging from 0.002–1.0 mg/mL Cells were incubated for 24 h at 37°C. Afterwards, 0.5 mg/mL of MTT was added, and the plates were incubated for additional 4 h. The formazan crystals produced by viable cells were dissolved in DMSO (100 μl) and subsequently shaken for 10 min at room temperature. The absorbance was measured at 570 nm using a Tecan Safire II (Crailsheim, Germany) spectrophotometric plate reader. The percentage cell viability was calculated using the following formula: % cell viability = (OD of treated cells / OD of control cells) x 100.

### Data analysis

All experiments were carried out in triplicate. IC_50_ value was determined as the concentration that resulted in 50% reduction in cell viability or inhibition of biological activity. IC_50_ values were calculated using a four parameter logistic curve using SigmaPlot 14.0, SYSTAT Software (CA, USA). Data were presented as mean ± standard deviation.

## Results and discussion

### GLC–MS analysis of the essential oils from different *Callistemon* species

Hydrodistillation of the fresh leaves of *C*. *citrinus*, *C*. *rigidus*, and C. *viminalis* yielded 0.43%, 0.84% and 0.41% w/w pale yellow essential oil, respectively. The identified components, their retention time, retention indices and percentages (average of three replicates for each species) for different seasons are summarized in Table 1. GLC/MS profiles of three *Callistemon* species collected during different seasons are displayed in supplementary material (S1–S4 Figs).

**Table 1:** Chemical profile of the essential oils of *Callistemon citrinus* (CC), *C*. *rigidus* (CR) and *C*. *viminalis* (CV) in four different seasons.

Peak no.	RT	Compound	RI_exp_^a^	RI_lit_^b^	Spring			Summer			Autumn			Winter		
					CC	CR	CV	CC	CR	CV	CC	CR	CV	CC	CR	CV
1	7.48	α-Thujene	920	923	0.04	0.02	0.09	0.03	0.67	0.04	0.26	0.02	0.07	0.05	0.01	0.94
2	7.68	α-Pinene	927	927	1.11	18.64	11.6	1.23	12.21	12.15	10.7	8.72	8.38	2.09	9.18	20.75
3	8.12	Camphene	943	943	0	0.06	0	0	0.02	0.02	0.01	0.02	0.02	0	0	0.02
4	8.9	β-Thujene	971	971	0.27	0	0.09	0.02	0	0	0	0	0	0.02	0	0
5	8.98	β-Pinene	974	974	0.8	1.21	1.13	0.86	0.65	1.09	0.58	0.67	1.36	1.42	0.51	0.73
6	9.44	β-Myrcene	991	991	1.19	0	1.06	1.36	0.30	0.25	0.17	0.02	0.70	1.76	0	0.05
7	9.73	2-Carene	1001	1001	0	0	0	0	0.03	0	0	0	0	0	0	0.07
8	9.83	Pseudo limonene	1004	1003	0.13	0.18	0.26	0.09	3.34	0.08	0.4	0.1	0	0.13	0.08	0
9	9.80	α-Phellandrene	1003	1003	0	0	0	0	0	0	0	0	0	0	0	0.87
10	9.98	3-carene	1009	1009	0	0	0	0	0	0	0	0	0	0	0	0.08
11	10.24	α-Terpinene	1017	1017	0.15	0	0.13	0.12	0.16	0	0	0	0	0.08	0	0.28
12	10.52	o-Cymene	1026	1026	0.10	0.42	0.28	0.15	3.67	0.47	1.78	0.25	0.96	0.31	0.25	5.7
13	10.54	D-Limonene	1025	1031	0	0	0	0	0	0	5.41	0	0	0	0	0
14	10.8	Eucalyptol	1035	1035	81.63	71.27	79.17	81.70	69.15	79.13	64.63	79.39	76.57	70.77	80	55.69
15	10.91	trans-β-Ocimene	1039	1040	0	0	0	0	0.05	0	0	0	0	0	0	0
16	11.24	cis-β-Ocimene	1049	1047	0.05	0.05	0	0.02	0.23	0	0	0	0	0	0	0.03
17	11.57	ɣ-Terpinene	1060	1060	0.65	0.5	0.76	0.62	0.76	0.46	0.2	0.21	0.61	0.72	0.15	0.71
18	12.5	Terpinolene	1090	1090	0.24	0.26	0.18	0.17	0.39	0.07	0.19	0.22	0.11	0.16	0.06	0.1
19	12.91	Linalool	1103	1103	7.61	0	0	7.78	0.34	0.18	0	0	0.36	13.80	0.17	0.76
20	13.33	Fenchol	1117	1117	0	0.05	0	0	0	0.03	0	0	0	0	0.05	0
21	14.13	trans-Pinocarveol	1142	1142	0	0.11	0	0	0	0.03	0.09	0.10	0.08	0	0.16	0.21
22	15.32	4-Terpinenol	1180	1180	1.17	0.39	0.69	1.40	0.63	0.72	0.28	0.55	1.41	1.61	0.42	1.01
23	15.74	α-Terpineol	1194	1193	2.84	4.59	2.13	3.353	6.14	3.63	10.27	5.72	4.82	4.27	5.69	3.4
24	17.6	Nerol	1258	1251	0.2	0.06	0.34	0.17	0.05	0.19	0	0.07	0.78	0.31	0.04	0.15
25	20.6	Eugenol	1363	1362	0.05	0	0	0.07	0	0	0	0	0	0.23	0	0
26	20.72	exo-2-Hydroxycineole acetate	1367	1367	0	0	0	0	0	0	0	0	0	0.10	0.09	0
27	21.23	Neryl acetate	1385	1385	0.09	0	0.11	0.11	0.11	0.06	0	0	0.24	0.13	0	0.04
28	22.34	*β*-Caryophyllene	1427	1427	0.14	0	0.15	0.03	0	0.03	0	0	0.17	0.07	0	0
29	26.42	*Spathulenol*	1586	1582	0	0	0	0	0	0	0	0	0	0.06	0.06	1.58
		**Monoterpene hydrocarbons**			4.76	21.30	15.62	4.70	22.51	14.67	19.72	10.26	12.25	6.78	10.26	30.37
		**Oxygenated Monoterpene**			93.47	76.47	82.35	94.42	76.48	83.92	75.29	85.84	84.04	90.76	86.62	61.38
		**Sesquiterpene hydrocarbons**			0.14	0	0.15	0.03	0	0.03	0	0	0.17	0.07	0	0
		**Oxygenated Sesquiterpene**			0	0	0	0	0	0	0	0	0	0.06	0.06	1.58
		**Others**			0.15	0	0.11	0	0	0	0	0	0.24	0.47	0.09	0.04
		**Total identified**			98.52	97.77	98.23	99.35	97.8	98.68	95.02	96.10	96.7	98.15	97.04	93.37

Compounds listed in order of their elution on RTX-5 GC column. Identification, was based on comparison of the compounds′ mass spectral data (MS) and retention indices (RI) with those of NIST Mass Spectral Library (2011), Wiley Registry of Mass Spectral Data 8^th^ edition and literature.

^*a*^ Retention index determined experimentally on RTX-5 column relative to n-alkane series (C8–C28)

^*b*^ Published retention indices.

Twenty-nine components were tentatively identified in the three *Callistemon* species. The results in Table 1 demonstrate that oxygenated monoterpenes followed by monoterpenes are the major oil components of the three species accounting for (61.38% - 94.42%) and (4.70% - 30.37%) of the total identified components, respectively. Meanwhile, sesquiterpenes and other classes were present in low abundance.

The major secondary metabolite of the three essential oils in different *Callistemon* species was eucalyptol (syn. 1,8-cineole) ranging from (71.27% - 81.70%), (69.15% -81.70%), (64.63% - 79.39%) and (55.69% - 80%) in spring, summer, autumn and winter, respectively, with the highest variation in summer and spring. Essential oils of the three *Callistemon* species in four seasons showed α-terpineol and α-pinene as major components in addition to eucalyptol. α-Thujene, β-pinene, O-cymene, ɣ-terpinene, terpinolene and 4-terpinenol were detected as common constituents in all *Callistemon* species.

Our results were consistent with previous reports on the essential oils obtained from the leaves of *Callistemon* species from different geographical regions. A study conducted on thirty Australian *Callistemon* species reported that 1,8-cineole was the major component (45–80%) of the majority of leaf essential oils. Other identified compounds include α-pinene (2–40%), limonene (2–9%) and α-terpineol (1–13%) [19]. Essential oil from the leaves of *C*. *lanceolatus* (syn. *C*. *citrinus*) from the north-eastern region of India exhibited 1,8 cineole (58.3%) as a major constituent followed by α-pinene, α-phellandrene, limonene and α-terpineol [20]. Likewise, the oil of *C*. *citrinus* from Reunion was found to be rich in 1,8-cineole (68.0%) followed by α-pinene and α-terpineol [21]. These results matched to a great extent the oil from the lower Himalayan region except for α-terpineol which was present at a lower percentage [22]. Essential oils from Brazil, were characterized by a high content of 1,8-cineole (77.0% and 65.0%) for *C*. *citrinus* and *C*. *viminalis*, respectively [23]. Essential oils from *C*. *viminalis* leaves were reported to possess 1,8-cineole (47.9%–82.0%) as the predominant constituent [24]. Similarly, oils of *C*. *citrinus* and *C*. *rigidus* from Cameroon were dominated by 1,8-cineole (73.8% and 79.1%, respectively) [25].

However, essential oils from *C*. *citrinus* leaves from Western Himalayas revealed high content of *α*-pinene (32.3%) followed by limonene (13.1%) and *α*-terpineol (14.6%), whereas, 1,8-cineole was only 9.8% of the leaf oil which controverted with previous studies from other geographical regions [26]. Thus, remarkable qualitative and quantitative variations in essential oil composition could be traced among plants collected in different geographical regions and /or seasons which necessitates construction of a simple and efficient chemometric model that could discriminate closely related species collected in different seasons.

### Discrimination of different *Callistemon* species by chemometric analyses

**Fig 1:**
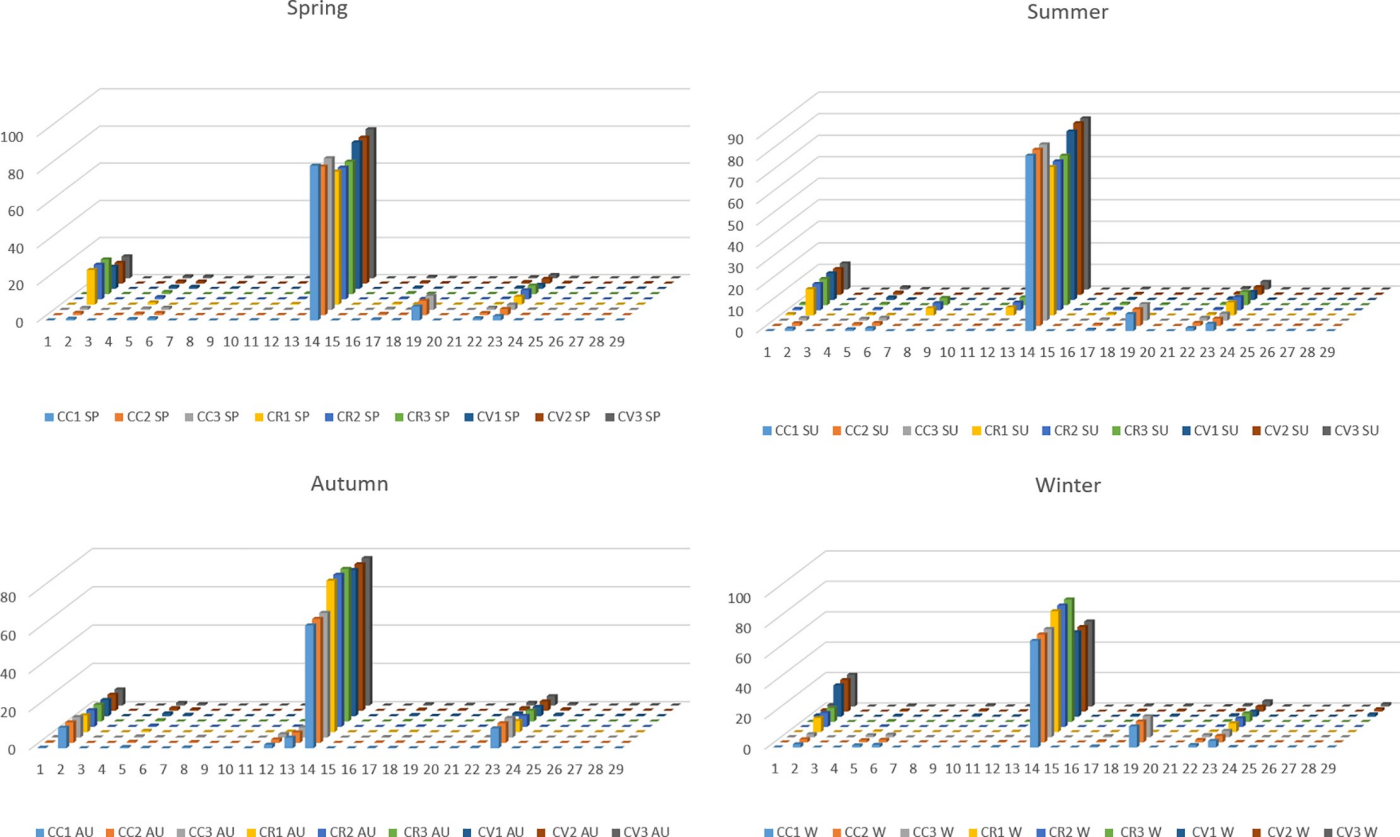
Bar charts of the main identified components of *Callistemon* species in spring, summer, autumn and winter.

Different bar charts were constructed for the major identified components of *Callistemon* essential oils. As shown in Fig 1, bar charts exhibited quantitative and qualitative differences regarding the metabolic profile of each species in each studied season. There are very close correlations between different *Callistemon* species in different seasons, as all samples showed eucalyptol as the major metabolite. Metabolic profiling (29 components, Table 1) were subjected to both PCA and HCA to reveal the chemical variability, and the inter-relationships between the oils in each season and among different species.

**Fig 2:**
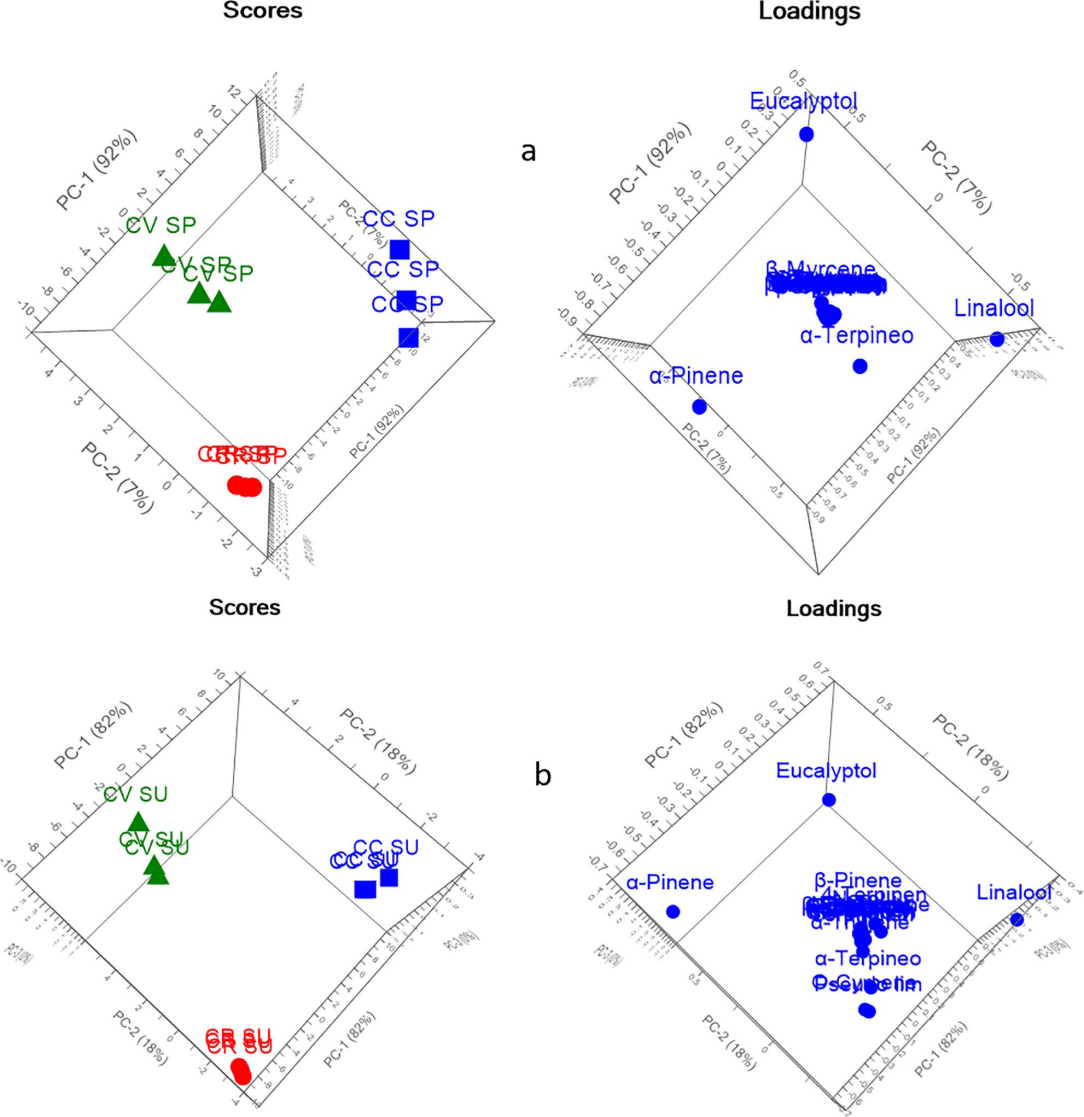
PCA score and loading plots of different *Callistemon* species (a) spring and (b) summer.

**Fig 3:**
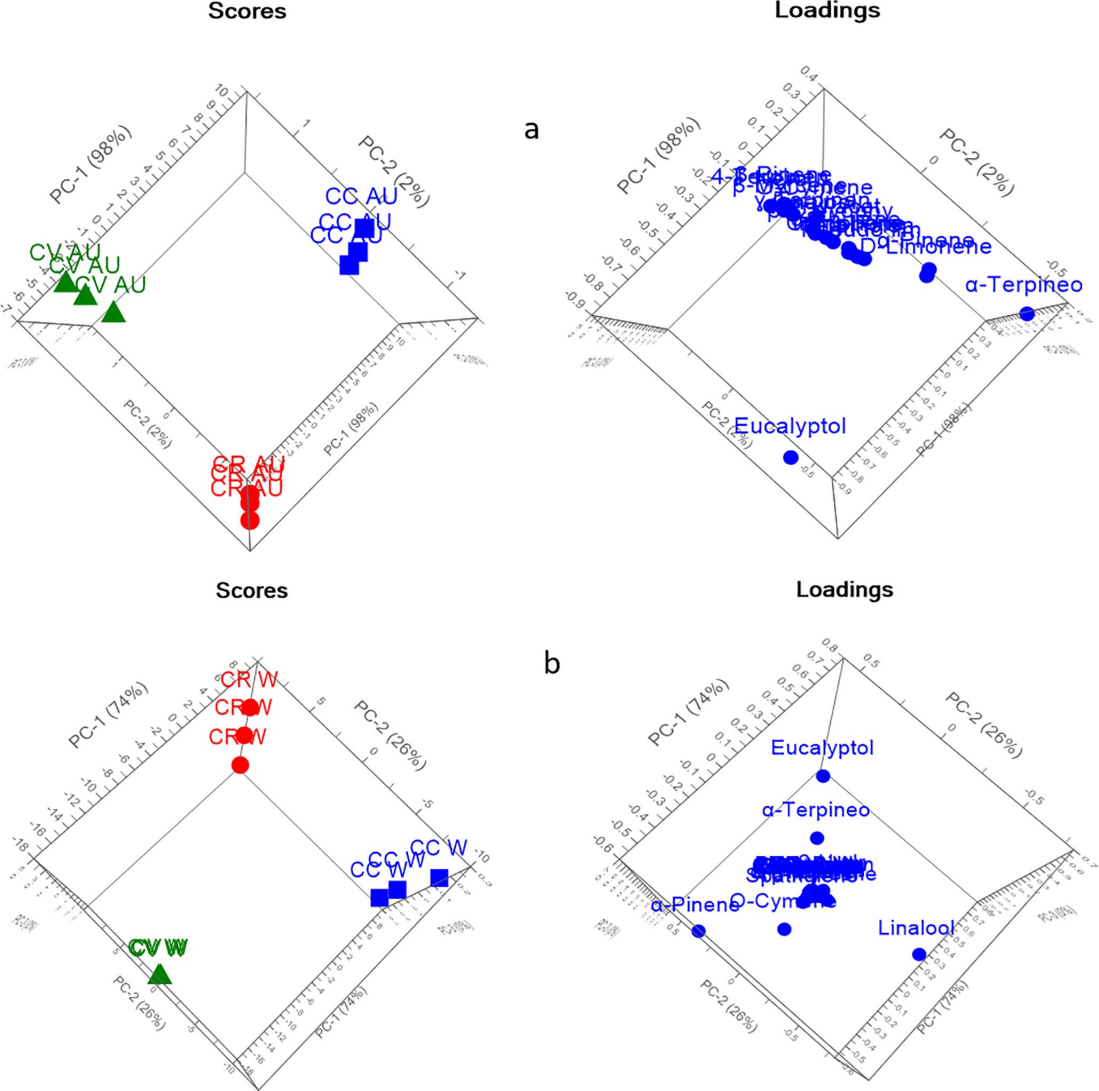
PCA score and loading plots of different *Callistemon* species (a) autumn and (b) winter.

PCA explained 99% and 100% of the variance of the data in spring and summer seasons, respectively, as shown in Fig 2A & 2B. The three species were significantly discriminated from each other, and each species was located in a different quadrant away from the other species. Loading plots showed that the main discriminating markers were eucalyptol, α-pinene and linalool. However, regarding autumn and winter season, PCA described about 100% of data discrepancy as presented in Fig 3A & 3B, where each species was completely segregated from each other. In addition to eucalyptol, α-pinene, and linalool, the loading plot showed that one main discriminating metabolic maker was α-terpineol, which highly influenced the segregation between the samples in winter season. However, for autumn both eucalyptol and α-terpineol were recognized as a marker for separation between different species.

**Fig 4:**
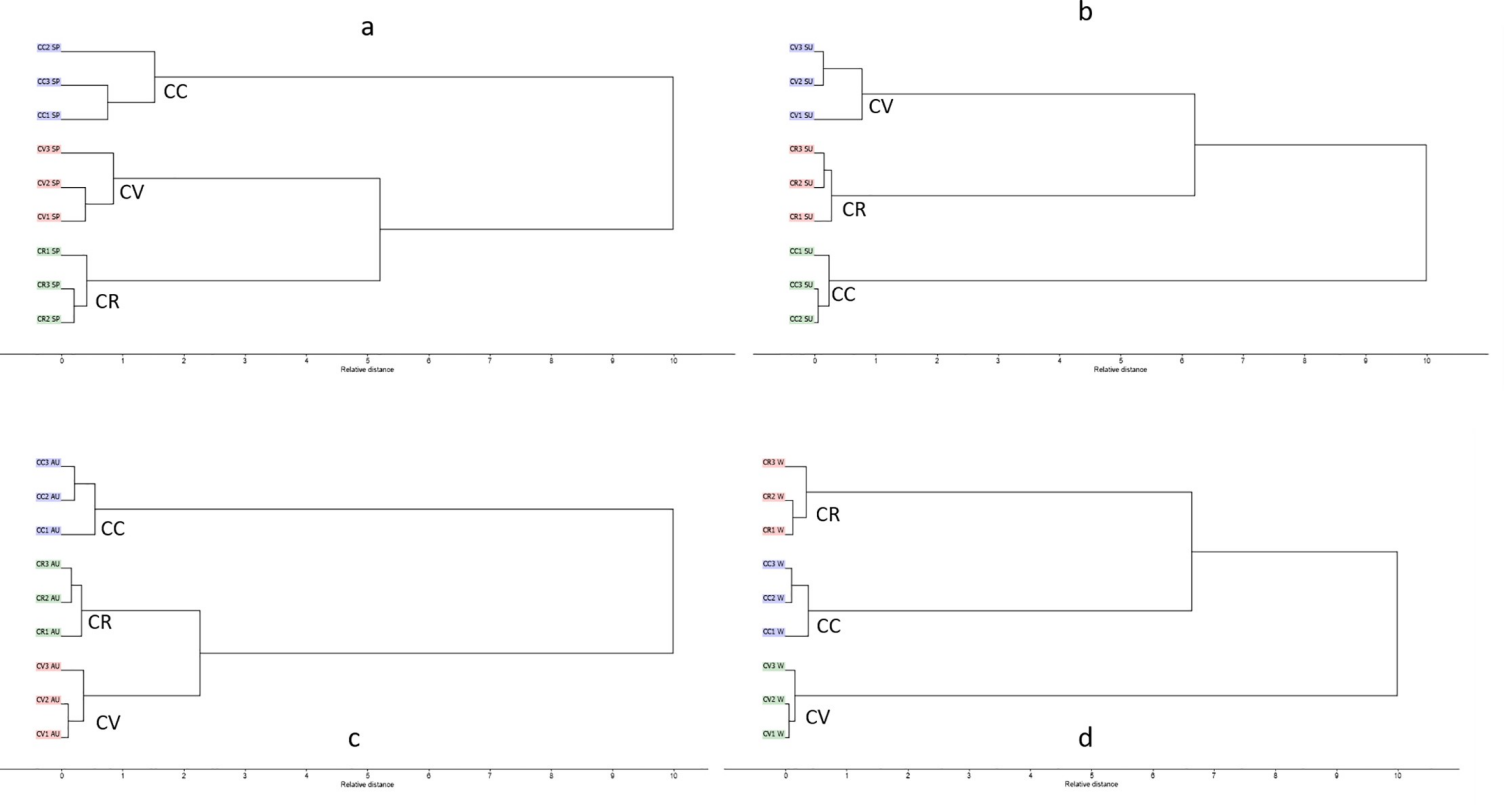
HCA of different *Callistemon* species (a) spring (b) summer (c) autumn (d) winter.

Additionally, HCA was applied as unsupervised pattern recognition method in order to confirm results obtained by PCA. The dendrograms obtained for different seasons, displayed in Fig 4, revealed segregation of different *Callistemon* species into three main clusters endorsing the results of PCA. HCA dendrograms revealed the near distance of *C*. *viminalis* and C. *rigidus* in spring, summer and autumn as presented in Fig 4A, 4B & 4C, respectively. On the other side, regarding winter season, HCA showed nearness of *C*. *citrinus*, *C*. *rigidus* in relation to *C*. *viminalis*.

**Fig 5:**
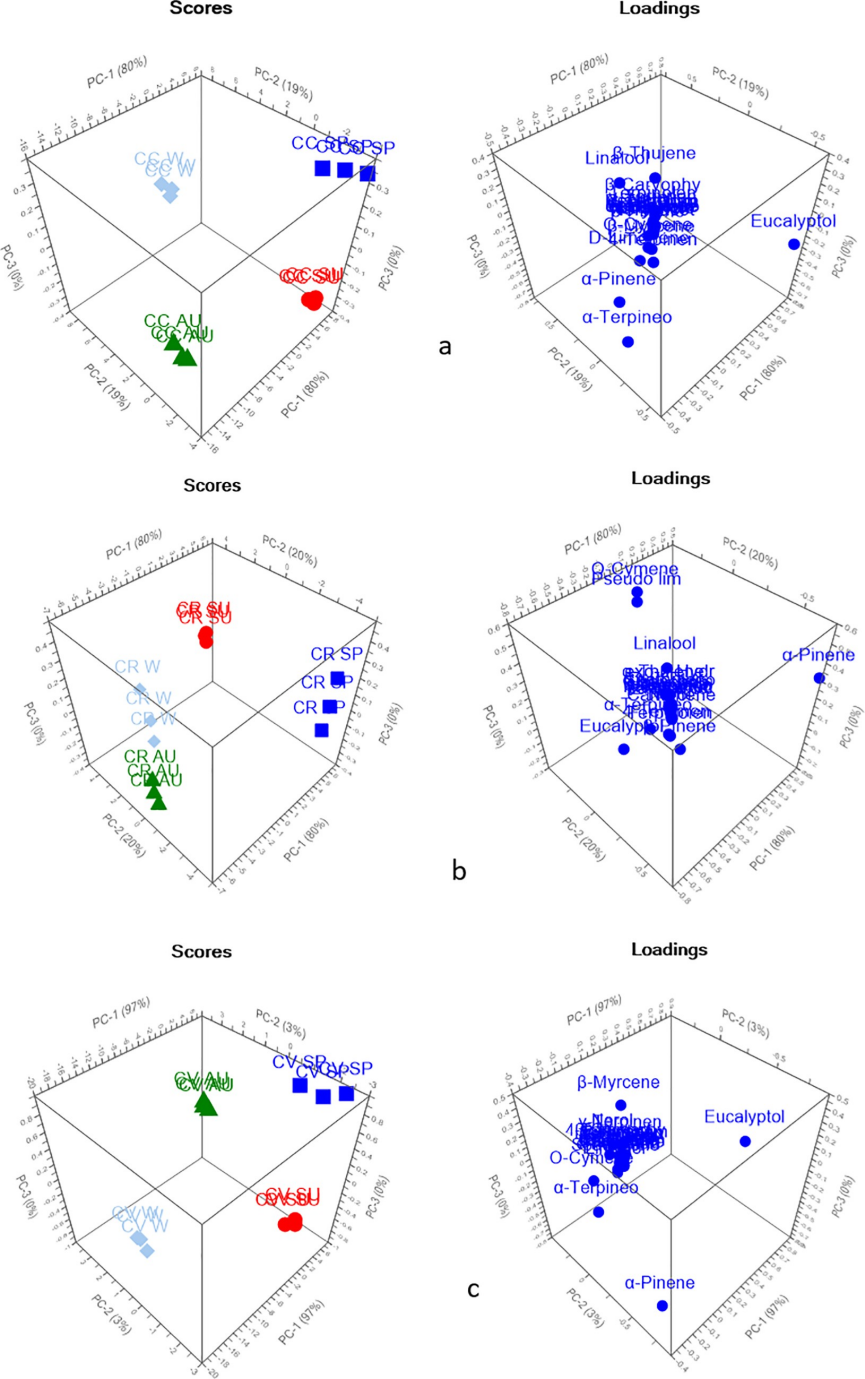
PCA score and loading plots of different *Callistemon* species (a) *C*. *citrinus* (b) *C*. *rigidus* (c) *C*. *viminalis* in all seasons.

In an attempt to find the relationship between the phytochemical profile of each *Callistemon* species in different seasons, PCA was applied as shown in Fig 5A, 5B & 5C. Regarding *C*. *citrinus* and *C*. *viminalis*, PCA score plot demonstrated the discrepancy in the chemical composition of the essential oils collected in each season where they were completely segregated from each other with eucalyptol, α-pinene and α-terpineol as major metabolites with the highest impact on the separation of *C*. *citrinus*. In addition to eucalyptol, α-pinene, β-myrcene exhibited an influence on the segregation of *C*. *viminalis* in different seasons. For *C*. *rigidus*, a substantial difference was observed between essential oils constituents in spring and summer that are distanced from each other, with regard to that of autumn and winter that are closely related. From the loading plot, it was found that *O*-cymene and pseudolimonene were the main markers responsible for the segregation of *C*. *rigidus* in summer, however α-pinene discriminates the species in spring.

**Fig 6:**
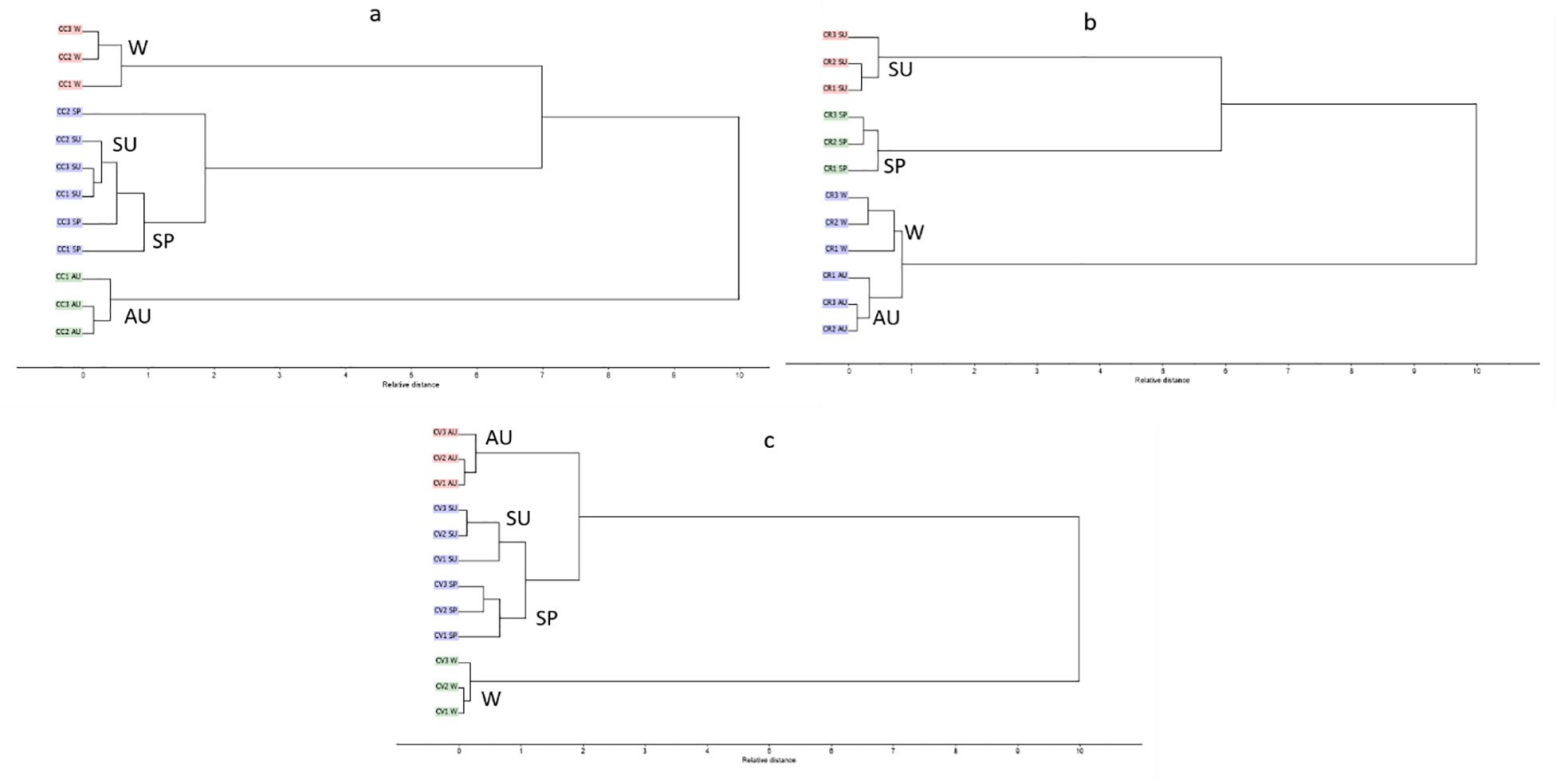
HCA of different *Callistemon* species (a) *C*. *citrinus* (b) *C*. *rigidus* (c) *C*. *viminalis* in all seasons.

HCA results for *C*. *citrinus*, *C*. *rigidus* and *C*. *viminalis* in different seasons are illustrated in Fig 6A, 6B & 6C. The resulting dendrograms displayed the same pattern as all showed three main clusters. Regarding *C*. *citrinus* and *C*. *viminalis*, the dendrograms revealed the close distance between spring and summer, as they are grouped in the same cluster. On the contrary, *C*. *rigidus*, indicated a close association between winter and autumn.

The essential oil composition of all *Callistemon* species in different seasons exhibited common major constituents as eucalyptol, α-terpineol and α-pinene, which make their discrimination a major obstacle. By applying metabolomics fingerprinting in combination with chemometric analysis, such as PCA and HCA, this problem could be solved and was helpful to identify the plants as it does not only rely on major components, but takes into consideration all metabolic profiling [27].

### Biological activity

#### Antioxidant activity

Essential oils have attracted attention for the plethora of bioactivities they possess. *Callistemon* essential oils were assessed for DPPH radical scavenging capacity. Antioxidant activity was presented herein as the concentration of essential oil that resulted in 50% free radical inhibition (IC_50_). *C*. *viminalis* showed more pronounced antioxidant activity than *C*. *citrinus*, exhibiting IC50 values of 1.40 mg/mL and 1.77 mg/mL, respectively. Nevertheless, *C*. *rigidus* showed very weak antioxidant activity with IC_50_ above the tested concentration range. Meanwhile, ascorbic acid exhibited IC_50_ value of 14.2 μg/ml. Results were in agreement with previous studies. Essential oil from the leaves of *C*. *citrinus* showed free radical scavenging activity with IC_50_ value of 4.02 mg/mL [28]. In another study, a pronounced free radical inhibitory activity (91.1 ± 0.3%) at a concentration of 1 mg/mL was observed for *C*. *citrinus* leaf essential oil, comparable to 0.1 mg/mL gallic acid (95.7 ± 2%) [29].

#### Anti-inflammatory activity

Inflammation is a normal defensive response to tissue injury or infection, functioning to combat invaders to remove damaged or dead host cells [30]. Erythrocytes membrane stabilization assay is considered a common tool to screen for anti-inflammatory candidates [17]. In this study, *Callistemon* essential oils showed inhibitory activity to the hemolysis of erythrocytes induced by hypotonic solution. *C*. *viminalis* showed potent membrane stabilizing activity exhibiting IC_50_ value of 25.6 μg/mL. Results were comparable to Indomethacin (IC_50_ 17.02 μg/mL). Moreover, *C*. *citrinus* showed moderate activity with IC_50_ value of 39.9 μg/mL. Meanwhile, *C*. *rigidus* displayed weak activity with IC_50_ value of 217.1 μg/mL.

The erythrocyte membrane is analogous to the lysosomal membrane. Thus, its stabilization serves as a parameter to assess the ability to stabilize the lysosomal membrane [17]. Stabilization of the lysosomal membrane is necessary to limit the inflammatory response through inhibition of the release of lysosomal constituents of activated neutrophils such as proteases and bactericidal enzymes. Exposure of erythrocytes to a hypotonic medium results in membrane lysis [31]. A possible mechanism for membrane stabilization activity of *Callistemon* essential oil observed herein could be attributed to the ability of essential oil constituents to integrate into cellular membranes, increasing the surface area to volume ratio of the cells that might be brought about by expansion of the membrane or shrinkage of the cell, as well as an interaction with membrane proteins [17]. Numerous terpenoids have been previously reported to possess anti-inflammatory activity. 1,8-cineole, was reported to inhibit the production of leukotrienes (LTB4) and PGE2 [32]. Furthermore, α-terpineol was reported to inhibit histamine release and reduce the production of inflammatory mediators [33]. Ahmed et al. reported that *C*. *citrinus* chloroform fraction exhibited membrane stabilizing anti-inflammatory potentials in hypotonic solution-induced hemolysis, results were comparable to acetylsalicylic acid, the standard drug [31].

#### Anti-proliferative Activity

The anti-proliferative activity of essential oils has been widely explored and numerous studies are now available in literature [10]. In this study, the anti-proliferative activity of *Callistemon* essential oils was assessed on HCT-116 and Hela human cancer cell lines using MTT assay. The three essential oils showed no cytotoxic activity. *C*. *citrinus* essential oil exhibited an IC_50_ value of 0.60 mg/mL on HCT-116 cancer cell line, whereas, IC_50_ values of 0.85 mg/mL and 0.51 mg/mL were recorded for *C*. *rigidus* and *C*. *viminalis*, respectively. Doxorubicin exhibited IC_50_ value of 4.62 μM on the aforementioned cancer cell line. On the other hand, the IC_50_ values recorded for *C*. *citrinus*, *C*. *rigidus* and *C*. *viminalis* on Hela human cancer cell line were 2.427, 3.428 and 1.928 mg/mL, respectively. These IC_50_ values indicate that the essential oils are not cytotoxic.

Previous studies conducted by Kumar *et al*. showed that *C*. *citrinus* leaf essential oil was not cytotoxic to rat glioma (C-6), human colon cancer (Colo-205), human cervical cancer (SiHa) and human peripheral blood mononuclear cells (PBMCs) at concentrations up to 100 μg/mL [26]. In the same context, essential oils obtained from *C*. *viminalis* leaves were not cytotoxic to melanoma cells (HT144) at concentration (200 μg/mL) where only 40% reduction in percentage cell viability was observed [34].

## Conclusion

The phytochemical profiling of the essential oils from three *Callistemon* species by GLC/MS showed that oxygenated monoterpenes represent the major class of the oil components with eucalyptol as the major secondary metabolite. The chemical profiles show high qualitative and quantitative similarities between species. Different chemometric analysis techniques were effectively applied as a discriminatory tool to differentiate between three *Callistemon* species, in each season, and within the same species in different seasons. *C*. *viminalis* essential oil exhibited pronounced membrane stabilization activity, which was equivalent to that of the standard drug, indomethacin. The three essential oils showed no cytotoxic activity against tested cancer cell lines. Future studies should be implemented to unravel other potential bioactivities of *Callistemon* essential oils.

## Supporting information

S1 Fig GLC/MS chromatograms of the essential oils of *C*. *citrinus* (CC), *C*. *viminalis* (CV) and *C*. *rigidus* (CR) collected in spring. (TIF)

S2 Fig GLC/MS chromatograms of the essential oils of *C*. *citrinus* (CC), *C*. *viminalis* (CV) and *C*. *rigidus* (CR) collected in summer. (TIF)

S3 Fig GLC/MS chromatograms of the essential oils of *C*. *citrinus* (CC), *C*. *viminalis* (CV) and *C*. *rigidus* (CR) collected in autumn. (TIF)

S4 Fig GLC/MS chromatograms of the essential oils of a *C*. *citrinus* (CC), *C*. *viminalis* (CV) and *C*. *rigidus* (CR) collected in winter. (TIF)
