# Supplementary figures and images for: Phytochemical profiling and seasonal variation of essential oils of three Callistemon species cultivated in Egypt
Source: PLoS One. 2019 Jul 11;14(7):e0219571. doi: 10.1371/journal.pone.0219571 (PMC6622538; doi:10.1371/journal.pone.0219571)

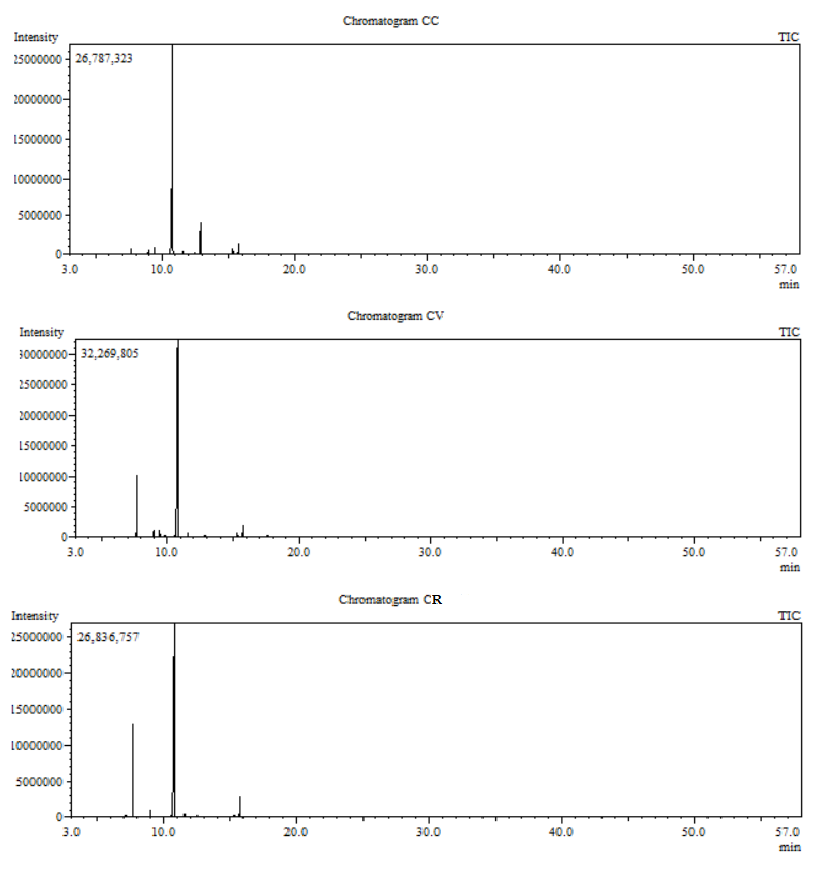

Supplement: S1 Fig — (TIF) [file pone.0219571.s001.tif]

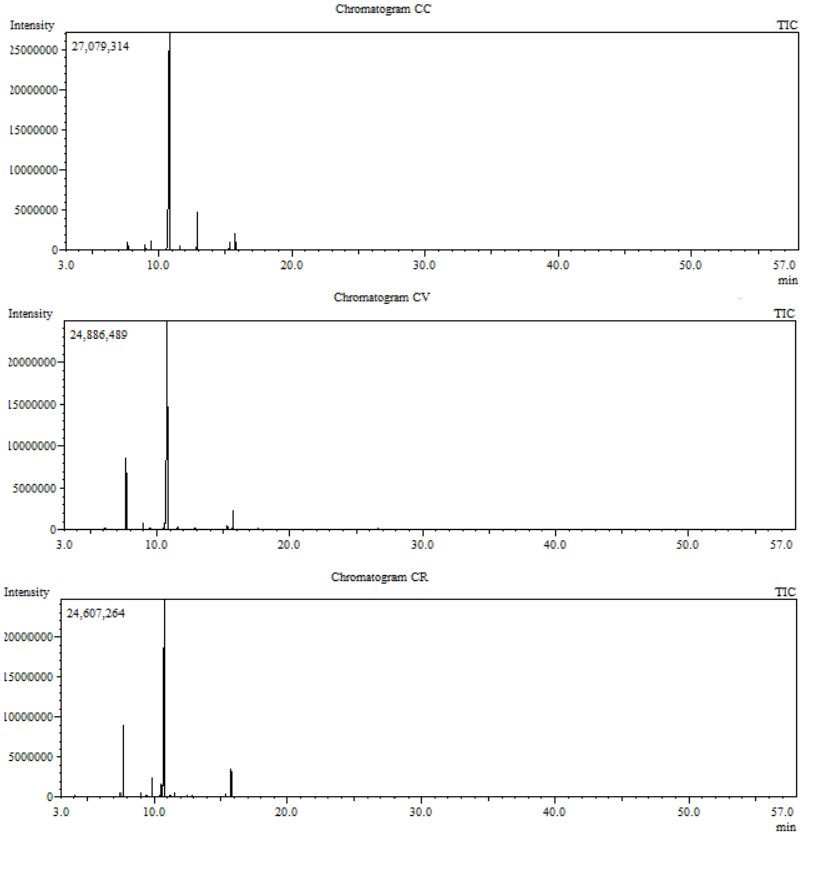

Supplement: S2 Fig — (TIF) [file pone.0219571.s002.tif]

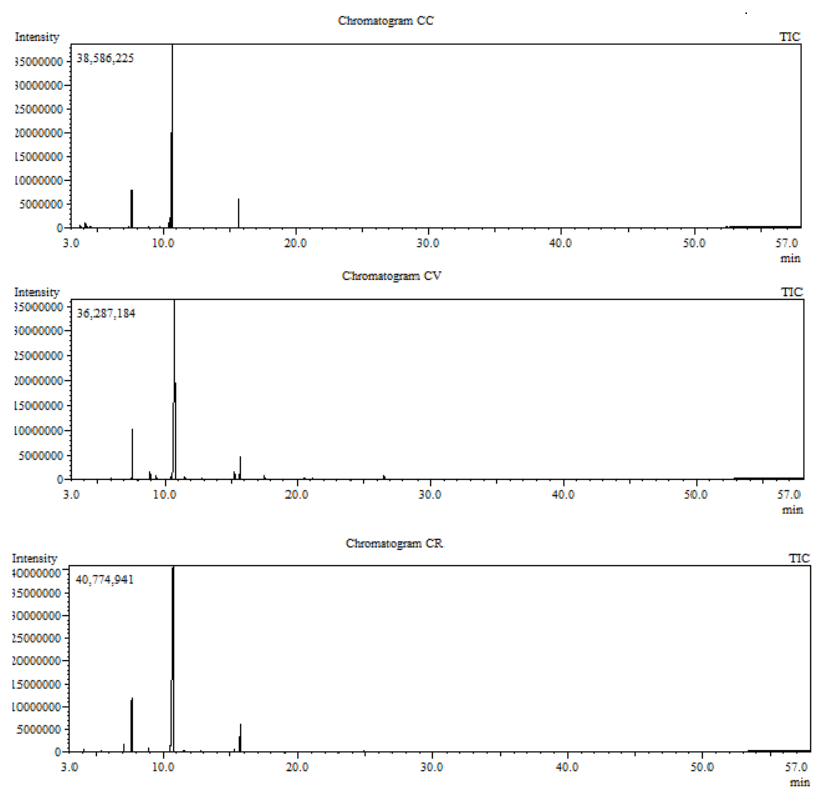

Supplement: S3 Fig — (TIF) [file pone.0219571.s003.tif]

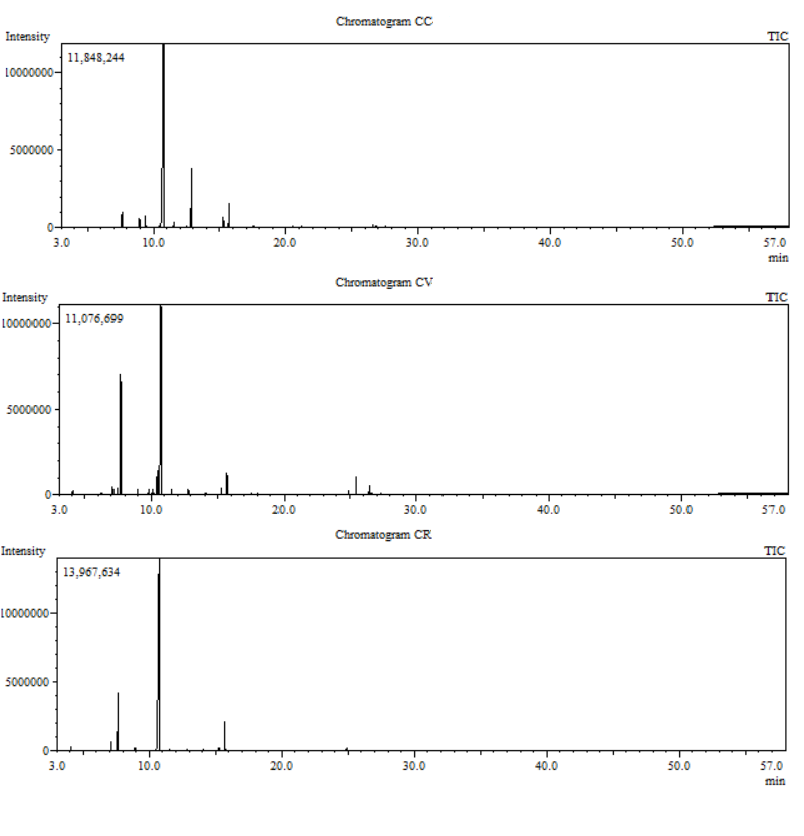

Supplement: S4 Fig — (TIF) [file pone.0219571.s004.tif]
